# Hopelessness Among Middle-Aged and Older Blacks: The Negative Impact of Discrimination and Protecting Power of Social and Religious Resources

**DOI:** 10.1093/geroni/igaa044

**Published:** 2020-09-15

**Authors:** Uchechi A Mitchell, Melissa Gutierrez-Kapheim, Ann W Nguyen, Nadia Al-Amin

**Affiliations:** 1 Division of Community Health Sciences, School of Public Health, University of Illinois at Chicago; 2 Jack, Joseph and Morton Mandel School of Applied Social Sciences, Case Western Reserve University, Cleveland, Ohio

**Keywords:** African American, Health and Retirement Study, Mental health, Religion and spirituality, Social networks and support

## Abstract

**Background and Objectives:**

Hopelessness—a state of despair characterized by a negative outlook towards the future and a belief in insurmountable challenges—is a risk factor for major depression, cardiovascular disease, and all-cause mortality among older adults. It is also an understudied consequence of discrimination. Older blacks disproportionately report experiencing discrimination and, as a result, may be at greater risk of feeling hopeless. However, social and religious resources may protect against the adverse effects of discrimination. The current study examines whether social support, social engagement, religious attendance, and religiosity buffer the effects of self-reported everyday discrimination on hopelessness among a nationally representative sample of blacks.

**Research Design and Methods:**

Using data from the 2010/2012 psychosocial assessment of the Health and Retirement Study, we regressed hopelessness on everyday discrimination, stratifying by 2 age groups, ages 51–64, representing middle-age (*n* = 1,302), and age 65 and older, representing old age (*n* = 887). Interaction terms tested whether each resource moderated the discrimination–hopelessness relationship controlling for depressive symptoms, socioeconomic status, and demographic characteristics.

**Results:**

Greater reports of everyday discrimination were associated with higher levels of hopelessness for middle-aged and older blacks. For middle-aged blacks, the resources did not moderate the discrimination–hopelessness relationship; rather, higher levels of support (*b* = −0.294, *p* < .01), religiosity (*b* = −0.297, *p* < .001), religious attendance (*b* = −0.218, *p* < .05) were independently and inversely associated with hopelessness. For older blacks, higher levels of religiosity moderated the discrimination–hopelessness relationship (*b* = −0.208, *p* < .05) and higher levels of support (*b* = −0.304, *p* < .05) and social engagement (*b* = −0.236, *p* < .05) were independently and inversely associated with hopelessness.

**Discussion and Implications:**

Findings suggest that self-reported everyday discrimination increases hopelessness among middle-aged and older blacks but social and religious resources may counterbalance its effects, in age-specific ways, to protect against hopelessness. Religiosity may be especially important for older blacks as a buffer against the negative consequences of discrimination on hopelessness.

**Translational Significance:** Self-reported everyday discrimination increases hopelessness among middle-aged and older blacks. Social and religious resources protect against hopelessness but do so differently for middle-aged and older blacks. The findings highlight the need for programs and initiatives that actively work to eliminate discrimination, foster supportive social ties, facilitate religious attendance, and foster greater religiosity to improve the mental well-being of blacks and help them hold on to hope despite the adversity they face.

## Background and Objectives

A large and multidisciplinary body of research has documented worse health among non-Hispanic blacks compared to non-Hispanic whites (1,2) and has identified the chronic stress of everyday occurrences of discrimination as a salient driver of health decline and accelerated aging among U.S. blacks (3). One pathway through which discrimination may influence the health of blacks is through increases in hopelessness (4). Individual and neighborhood-level stressors are associated with hopelessness (5–8). Hopelessness, in turn, is associated with several adverse health outcomes that tend to be more prevalent in black communities (9,10) and are often attributed to their chronic experiences of discrimination (3). Thus, it is plausible that experiences of discrimination may be an important risk factor for hopelessness among blacks, warranting examination of their association and the identification of protective factors.

## Conceptualizations of Hopelessness

For nearly 60 years, researchers across disciplines have studied hopelessness and its adverse effects on health and functioning (11–13). Hopelessness is a cognitive state characterized by negative expectations towards the future and the belief that one cannot achieve sought after goals (12,14). Research on its psychological counterpart, hope, further suggests that the absence of strategies or plans for achieving one’s goals might also be characteristic of hopelessness (15). Current research, however, is equivocal on whether hope and hopelessness are on opposing ends of a single construct (13,16). Nonetheless, hopelessness conceptually combines theoretical and empirical research on generalized outcome expectancies (17) with research on self-efficacy and helplessness (18,19); it may also incorporate research on goal-oriented thinking and motivation (20). In this way, hopelessness is distinct from concepts such as optimism, which is solely defined by one’s outlook towards the future (21), and internal sense of control, which focuses on personal agency regardless of one’s expectations for the future (22).

Early research on hopelessness considered it to be a symptom of depression; specifically, it was described as the “missing link” explaining why some depressed individuals attempted suicide while others did not (11,12). This perspective of hopelessness aligns with the *Diagnostic and Statistical Manual of Mental Disorders’* criteria for diagnosing depressed mood among adults (23). However, others have argued that hopelessness may be an antecedent and subtype of depression resulting from negative life events and other stressors (14). This “Hopelessness Theory of Depression” has gained prominence over the years. Several studies have shown that hopelessness is predictive of depression and that a person can be depressed without being hopeless and hopeless without being depressed. Thus, hopelessness and depression are distinct constructs that independently predict health (24).

## Hopelessness and the Stress of Discrimination for U.S. Blacks

The health significance of hopelessness cannot be overstated. In addition to an increased mortality risk (9), hopelessness is associated with the refusal of life-sustaining treatment among the chronically ill (25) and suicidal thoughts and attempts (6,26). In other words, hopelessness may contribute to a loss of the will to live that is replaced by a desire to die. For this reason, identifying populations at greatest risk for hopelessness and its determinants is of high priority. Prior research has shown that older adults and individuals who are socially and economically disadvantaged are at greater risk for feeling hopeless. For example, Mitchell and colleagues (7) found that older adults with lower levels of educational attainment were more likely to be hopeless and to experience greater increases in hopelessness as they aged compared to those with higher levels of education. Others have similarly found that hopelessness is associated with life course socioeconomic status (27), living in socioeconomically disadvantaged neighborhoods (5) and more frequent exposure to stressors (6,7). Given research indicating lower educational attainment, greater social and economic disadvantage, and more frequent stress exposure among blacks (28), the effects of hopelessness on the health of black Americans have increasingly become of interest. Most of this research has focused on how hopelessness impacts the health of black college students and black adolescents living in impoverished communities (29,30). Research on the effects of racism and discrimination on hopelessness among middle-aged and older blacks is scarce, despite aging, cohort, and life course differences that potentially shape the relationship between discrimination and hopelessness in unique ways. Therefore, there is a need to better understand the impact of experiences of discrimination on feelings of hopelessness among middle-aged and older blacks.

Blacks and other racial and ethnic minorities disproportionally report experiencing discrimination (31,32), which negatively impacts their mental health and overall well-being (33–36). Theoretical frameworks on racism and discrimination are informed by social stress theory and often mention hopelessness as one of the many deleterious consequences of discrimination (4). However, few have assessed this conceptual assertion. As an interpersonal stressor, discrimination is defined as unfair, differential treatment that disadvantages one group over another based on one or more personal characteristics (37). The stress of discrimination stems from it being an unavoidable, uncontrollable, unpredictable, and unfair experience that denigrates and demeans a core aspect of one’s identity. In this way, repeated experiences of acute and chronic forms of discrimination can erode a sense of self and contribute to feelings of powerlessness and, ultimately, hopelessness. That said, the stress process model suggests that the extent to which a stressor adversely affects a person’s mental health is intricately tied to the resources available to cope with the stressor (38). Therefore, the impact of discrimination on hopelessness should be examined in the context of available social and psychological resources.

## The Role of Social and Religious Resources

The social and psychological resources black Americans use to cope with discrimination and adversity are uniquely tied to their lived experiences. Social engagement and emotional support from family and friends are two such resources. Although there is great diversity in the experiences of blacks in the United States, sociological and historical forces have shaped the black family and social network in distinct ways (39,40). The presence of intergenerational households, large and well-integrated extended family relationships, “church family,” and fictive kin (i.e., close non-family ties) are common elements of their social support networks and often serve as resources that protect against the psychological distress of racism and other forms of oppression (41). Research on social connectedness, or engagement, among blacks has shown that they are well integrated within their social networks (42) and highly involved in supportive exchanges, both frequently receiving and providing support to network members (43). Moreover, rates of social engagement are high among blacks (42), as they are in frequent contact with their family, friends, and church members (42,44).

The social support and engagement derived from these relationships are critical for coping with stress and protecting against mental health conditions. Extant research has shown that blacks who receive greater emotional support from their family are less likely to have depression (45), social anxiety disorder (46), posttraumatic stress disorder (47), and suicidal ideation and attempts (44). Although negative interactions with family members or friends can be a source of stress for blacks, the emotional support received can lessen the impact of these interactions to limit adverse mental health (48).

Religious beliefs, regular religious service attendance, and church members are also important resources in the black community. Historically, the Black Church has been a foundational institution in the lives of black Americans (49). A tradition of research has documented the importance of the Black Church not only as a religious institution but also a cultural, social, educational, financial, and political institution that is central to many black communities. For example, the Black Church was an important symbol of the Civil Rights Movement and some of the most prominent leaders of this movement, such as Martin Luther King, Jr., Fred Shuttlesworth, and Ralph Abernathy, were clergymen. Black churches were major points of mobilization for the effort as church members provided food and housing to civil rights workers and comprised a great proportion of the participants in civil rights demonstrations. In addition to their involvement in the Civil Rights Movement, throughout history, black churches have offered and still offer a wide range of community outreach programs and services, including senior citizen groups, antipoverty and material aid programs, food and clothing banks, health services, tutoring and educational programs, and employment programs (49).

Given the integral role religion and church have played in the lives of black Americans, it is not surprising that older blacks, as a group, have the highest level of religious involvement (e.g., service attendance, prayer) and subjective religiosity (i.e., religious attitudes) (50) even compared to younger blacks (51). These patterns of religious involvement, religiosity, and the significance of the Black Church denote the importance of religion as a psychological resource for older blacks. Additionally, empirical research has shown that religious involvement can protect against mental health problems and promote well-being (52). Research on religiosity indicates similar protective qualities for blacks; specifically, religiosity protects against psychological distress and promotes happiness and life satisfaction (53).

Altogether, research on social and religious resources in the black community has shown that they directly protect against poor mental health outcomes. Some evidence also exists for their role as buffers against the negative mental health effects of stress and discrimination (48,54). However, what is largely absent in the literature is research on whether they buffer the effects of discrimination on hopelessness, specifically. Some research suggests that these resources have a direct protective affect against hopelessness. For instance, Panzarella and colleagues (55) showed that social support promoted more adaptive inferences of negative life events that reduced feelings of hopelessness. Additionally, Cruz and colleagues (56) showed that prayer and meditation were associated with reduced odds of being hopeless and Murphy and colleagues (57) showed that religious beliefs were associated with lower levels of hopelessness. Both studies, however, did not find an association between church attendance and hopelessness.

Although some research exists on the interrelations between discrimination, social and religious resources and mental health, there is only one, to our knowledge, that specifically examined hopelessness. Odafe and colleagues (8) examined the relationship between race-related stress—a measure evaluating discrimination at the institutional, cultural and individual levels—hopelessness and different types of interpersonal support. They found a positive association between race-related stress and hopelessness and a significant interaction between race-related stress and self-esteem-specific social support: among those with low support levels, race-related stress was associated with greater hopelessness, but among those with high levels of support there was no association. This study, however, was limited to blacks living in the South and excluded older adults 65 and older. Thus, there is some—but limited—empirical evidence to suggest that discrimination is a risk factor for hopelessness and that social resources, like social support, mitigate its effects. The role of religious resources, however, is unclear.

## The Current Study

The current study addresses the stated gaps in the literature by assessing the effects of discrimination on hopelessness in a nationally representative sample of blacks in midlife and old age. We also assess the protective role of multiple social and religious resources, specifically, social support, social engagement, religious service attendance, and religiosity, to identify factors that diminish hopelessness among both middle-aged and older blacks when they are faced with discrimination.

Empirical evidence indicates that age is a confounding variable in the aforementioned relationships between discrimination, social and religious resources, and hopelessness and argues for an age-stratified analysis to account for its confounding effects. First, age is positively associated with hopelessness (58) and inversely associated with reports of everyday discrimination (35,59); older blacks are more likely to be hopeless but report less frequent experiences of everyday discrimination than middle-aged blacks. The inverse association between age and discrimination may indicate a cohort effect, as the current cohort of older blacks came of age before the Civil Rights Act of 1964 and experienced more explicit and hostile forms of discrimination compared to their middle-aged counterparts, who mostly came of age after its passage. Because everyday discrimination taps into daily hassles and hostilities (i.e., microaggressions) older blacks may not identify these experiences as discriminatory. In addition to differences in the frequency of reported discriminatory experiences, studies have demonstrated that experiences of discrimination are qualitatively different between middle-aged and older blacks (59,60). Second, the heterogeneity in the distribution of social resources over the life course necessitates an age-stratified analysis. Research on social relationships indicates that social network size and social support exchanges decrease with age, such that older adults tend to have smaller social networks (61) and receive less support than middle-aged adults (62). Thus, the availability and function of social relationships vary across age groups, and findings in this area suggest that their stress coping functions operate uniquely for middle-aged and older adults (63). Third, religiosity and religious involvement increases with age. Older blacks attend religious services more frequently, have higher levels of subjective religiosity, and attribute greater importance to religion in their lives than their younger counterparts (64). Given its importance among older blacks, religion may be a more relevant and readily accessible coping resource for older blacks than middle-aged blacks. In fact, research has demonstrated that older blacks are more likely to engage in religious coping than younger blacks (65). Taken together, this body of evidence suggests that age is a confounding variable, which warrants separate examination of the nature of these associations in middle-aged and older blacks to better isolate the effects of discrimination and social and religious resources on hopelessness.

We hypothesize that self-reports of everyday discrimination will be positively associated with hopelessness and that each of the social and religious resources will be inversely associated with hopelessness for both age groups. However, given the previously discussed developmental differences between middle-aged and older adults, we hypothesize that social resources, a more developmentally relevant coping resource for middle-aged adults, will fully buffer the effects of discrimination on hopelessness among middle-aged blacks (i.e., there will be a null discrimination–hopelessness association in the high social support and social engagement groups) and partially buffer against the effects of discrimination among older blacks (i.e., there will be a significant but less pronounced discrimination–hopelessness association in the high religious attendance and religiosity groups). Conversely, we hypothesize that religious resources, a more developmentally relevant coping resource for older adults, will partially buffer the effects of discrimination among middle-aged blacks and fully buffer the effects of discrimination among older blacks.

## Research Design and Methods

### Sample

We used data from the Health and Retirement Study (HRS), a nationally representative panel survey of the health and aging experiences of community-dwelling adults aged 51 and older, living in the contiguous United States (66). The HRS began in 1992 and repeats data collection on core survey topics every 2 years. Initial interviews are conducted face-to-face while follow-up interviews occur by telephone. In 2006, a random half-sample of the HRS cohort was given a self-administered, leave-behind questionnaire on psychosocial factors. The other half-sample completed the same assessment in 2008. Each half-sample is followed up every 4 years. In the current study, we combined data from the 2010 and 2012 half-samples to increase our statistical power and restrict our analyses to respondent who self-identify as non-Hispanic black. There were 14,714 eligible HRS respondents in 2010/2012. Of this sample, 2,346 (16%) self-identified as black and 2,189 (93%) had complete data on all study measures. Respondents with missing data (*n* = 157) were more likely to be male, have less than a high school degree, live below the poverty line, not be employed, be widowed, have lower levels of social engagement, and attend religious services less often. The HRS was approved by the University of Michigan’s Institutional Review Board (IRB) and because the present study used de-identified, publicly available data, the University of Illinois, Chicago’s IRB deemed the study exempt from review.

### Study Measures

#### Hopelessness

Hopelessness was measured using four items. Two items came from Everson and colleagues (67): “I feel it is impossible for me to reach the goals that I would like to strive for” and “The future seems hopeless to me and I can’t believe that things are changing for the better.” The other two items came from Beck and colleagues (12): “I don’t expect to get what I really want” and “There is no use in trying to get something I want because I probably won’t get it.” Possible responses ranged from 0 = “strongly disagree” to 5 = “strongly agree.” Factor analyses revealed a single-factor structure for the four items and the Cronbach’s α was .83 and .85 for the 2010 and 2012 half-samples, respectively. Responses to a minimum of two of the four items were needed to construct the scale, which we averaged such that higher scores represented greater feelings of hopelessness.

#### Everyday discrimination

Everyday discrimination refers to unfair treatment that manifests as daily slights and indignities in everyday settings. Six items from the Everyday Discrimination Scale were used to assess the frequency of reported experiences of this interpersonal form of discrimination (36). The items asked respondents about the following experiences: “You are treated with less courtesy or respect than other people,” “You receive poorer service than other people at restaurants or stores,” “People act as if they think you are not smart,” “People act as if they are afraid of you,” “You are threatened or harassed,” and “You receive poorer services or treatment than other people from doctors or hospitals.” Possible response options ranged from 0 = “never” to 5 = “almost every day.” This measure of discrimination is not specific to a type of discrimination and therefore represents a general measure of everyday discrimination regardless of attributions or reported reasons why discrimination occurred (e.g., race, gender, age, etc.). In line with previous psychometric studies of this measure (68), a single-factor structure existed for the six items. The Cronbach’s α was .80 and .81 for the 2010 and 2012 half-samples, respectively, and higher values represented more frequent reports of everyday discrimination.

#### Social and religious resources

Two measures each were created to assess the protective effects of social and religious resources on hopelessness, generally, and on the effects of the discrimination–hopelessness relationship, specifically. All four measures were dichotomized at their respective medians to facilitate interpretation of study findings. Sensitivity analyses with continuous versions of the resource variables did not change the substantive findings of the study in a meaningful way.

##### Social support.

—Emotional social support refers to the care, empathy, trust, and acceptance offered to another (69). Three items each were used to assess the extent to which a respondent receives emotional support from a spouse/partner, child(ren) family members, and friends. The three items include: “How much do they really understand the way you feel about things,” “How much can you rely on them if you have a serious problem,” and “How much can you open up to them if you need to talk about your worries.” Responses ranged from 0 = “not at all” to 3 = “a lot.” We averaged all the items and dichotomized the scale at its median (i.e., 2) to differentiate low (reference category) versus high social support.

##### Social engagement.

—Social engagement is a measure of contact and connection with a person’s family, friends, and neighbors. In line with previous work (70), we assessed five dichotomous indicators of social engagement: weekly contact with parents, weekly contact with children, weekly contact with friends, weekly contact with neighbors, and at least 1 hr of volunteering in the past year. A count of the social engagement experiences was created, ranging from 0 to 5, and then dichotomized at its median (i.e., 3) to differentiate low (reference category) from high social engagement.

##### Religious service attendance.

—Regular attendance at religious services may facilitate connection and mutual support among attendees and provide messages that help develop or sustain internal personal resources. Respondents were asked: “About how often have you attended religious services during the past year.” We compared respondents who infrequently attended religious services (i.e., less than once a week; reference category) to those who attended at least once a week or more. We dichotomized religious attendance in this manner because prior research suggests that most Americans in midlife and old age attend a religious service at least weekly (71). Moreover, 53% of our study sample reported weekly or greater religious service attendance.

##### Religiosity.

—Religiosity refers to the extent to which a person believes in a higher power and receives guidance and comfort from this belief. Four items assessed religiosity: “I believe in a God who watches over me,” “The events in my life unfold according to a divine or greater plan,” “I try to carry my religious beliefs over into all my other dealings in life,” and “I find strength and comfort in my religion.” Response options ranged from 0 = “strongly disagree” to 5 = “strongly agree.” We averaged the four items to construct the religiosity scale and then dichotomized the scale at its median (i.e., 5) to distinguishes between high and low levels of religiosity. Those with a mean value equal to 5 were included in the high religiosity group, and those with a mean value less than 5 were included in the low religiosity group (reference category).

#### Covariates

All analyses were stratified by two age groups: ages 51–64 years old represented respondents in midlife and ages 65 and older represented respondents in old age. Gender, marital status, and nativity were included as demographic predictors in the model. Females are compared to males (reference category); individuals who were married or cohabiting (reference category) were compared to those who were divorced or separated, widowed, and never married; and foreign-born individual are compared to U.S.-born individuals (reference category).

We also included measures of socioeconomic status because they have been shown to be associated with experiences of discrimination and hopelessness. Individuals with less than a high school degree (reference category) were compared to those with a high school degree, some college, and a college degree or higher. Employed individuals (reference category) were compared to individuals who were partly retired, retired, and not in the labor force, and individuals with household incomes at or below the Federal Poverty Line for 2010/2012 (reference category) were compared to those living above the poverty line.

We included a measure of depressive symptoms in our analyses because it is associated with everyday discrimination (72) and hopelessness (24) and therefore may confound this relationship. We used an abbreviated eight-item version of the Center of Epidemiological Studies Depression Scale (73) that was modified from a scaled response format to a yes-no response format. The eight items assessed include: “felt depressed,” “everything is an effort,” “sleep is restless,” “felt alone,” “felt sad,” “could not get going,” “felt happy” (reverse coded), and “enjoyed life” (reverse coded). A count variable was then created, ranging from 0 to 8. Higher values indicated more depressive symptoms.

### Statistical Analyses

We began our analyses by examining weighted descriptive statistics—means, standard errors, percentages, and sample size—of all study variables for each age group (i.e., middle-aged and old age). Multiple linear regression was used to evaluate the relationship between hopelessness and everyday discrimination for middle-aged and older blacks, separately. Model 1 regressed hopelessness on everyday discrimination and demographic and socioeconomic covariates. Model 2 added the four resources: social support, social engagement, religious attendance, and religiosity. The next four models (i.e., Models 3–6) separately assessed the interaction between discrimination and each of the four resources to determine whether social support, social engagement, religious attendance, and religiosity function as moderator of the relationship between discrimination and hopelessness. Supplemental analyses examined the distribution of discrimination attributions and regressed hopelessness on an indicator for whether or not a respondent mentions race as a reason for their experiences of discrimination and its interaction with everyday discrimination. These analyses were done to determine if the study findings differed when a general measure of discrimination is used compared to a race-specific measure. All analyses used Stata version 15.1, weights provided by the HRS and survey procedures in Stata to account for the complex sample design of the HRS.

## Results

Weighted sample characteristics are presented in Table 1 for respondents middle-aged (age 51–64; *n* = 1302) and older (age 65+; *n* = 887). Although the full range of values for hopelessness were seen in both age groups, approximately, 26.8% of respondents strongly disagreed with all items of the hopelessness scale and had a hopelessness score of zero. Mean levels tended to be low and were equivalent to strongly or somewhat disagreeing (i.e., response options between 0 and 1) with statements characterizing hopelessness. Similarly, reports of everyday discrimination were relatively low for middle-aged and older blacks. Thirty-three percent of respondents reported “never” experiencing any of the discriminatory experiences assessed in the scale and average values were equivalent to reporting never experiencing discrimination (i.e., response option of 0) or experiencing it less than once a year (i.e., response option of 1). For middle-aged blacks, two-thirds or more had high levels of social support and social engagement, slightly more than 50% had high religiosity levels, and a little less than half attended a religious service weekly. For older blacks, 60% or more of respondents had high levels of each social and religious resources.

**Table 1.** Weighted Sample Characteristics among Blacks Age 51–64 and 65+: Health and Retirement Study, 2010/2012 (*n* = 2,189)

	Blacks Age 51–64 (*n* = 1,302)		Blacks Age 65+ (*n* = 887)	
	Mean or %	*n*	Mean or %	*n*
Key variables				
Hopelessness (range: 0–5)^a^	1.3	1,302	1.5	887
Everyday discrimination (range: 0–5)^a^	0.9	1,302	0.6	887
High social support	62.9	819	71.4	633
High social engagement	65.8	857	70.0	619
High religiosity	51.2	667	60.3	535
Weekly religious service attendance	48.4	630	58.9	522
Additional covariates				
Age (range: 51–101)^a^	57.2	1,302	74.0	887
Female	66.7	868	65.3	579
Foreign-born	6.4	83	6.3	56
Married/partnered	46.3	603	45.0	399
Divorced/separated	28.5	371	18.5	164
Widowed	10.1	132	31.3	278
Never married	15.1	196	5.2	46
Less than high school	16.5	215	33.4	296
High school degree	33.3	433	34.2	303
Some college	33.3	433	20.5	182
College graduate	17.0	221	12.0	106
Employed full-part-time	49.2	640	7.4	66
Partly retired	5.1	66	10.8	96
Retired	31.3	407	76.4	678
Unemployed/not in labor force	14.5	189	5.3	47
Depression (range: 0–8)^a^	2.0	1,302	1.8	887

*Note*: ^a^Range in the entire sample of blacks (age 51+).

With regard to sociodemographic characteristics, two-thirds of middle-aged blacks were female and nearly 50% were married or partnered. An approximately equal proportion had at least a high school degree or more. Close to 50% were employed and on average middle-aged blacks reported two depressive symptoms. Two-thirds of older blacks were female and while 45% were married, nearly one in three older blacks were widowed. More than two-thirds had a high school degree or less and only 7% were employed. Older blacks also reported two depressive symptoms, on average.

**Figure 1.**
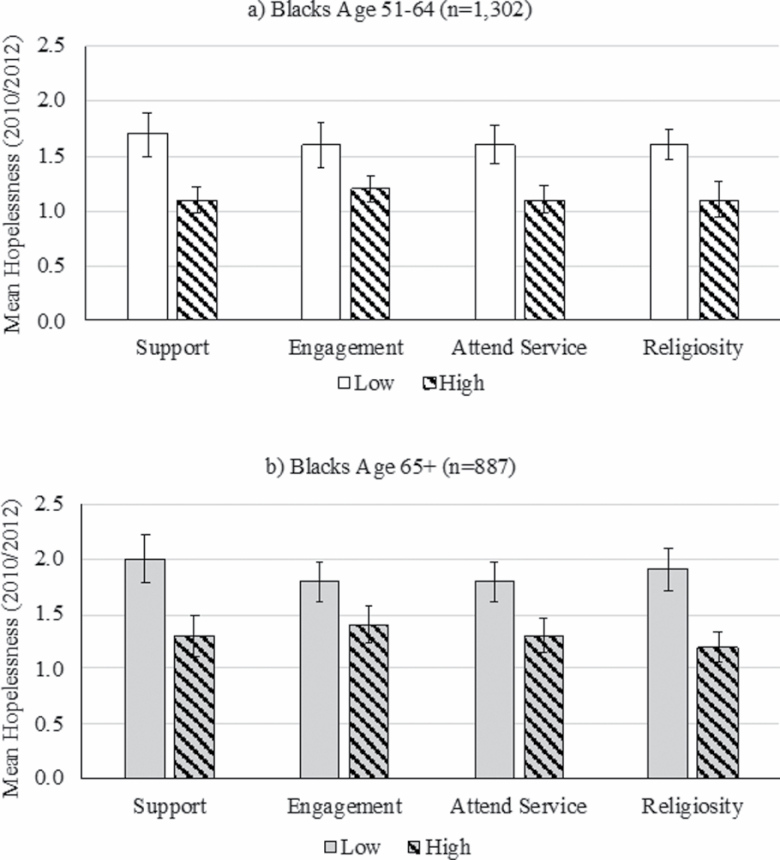
Weighted mean levels of hopelessness among blacks age 51–64 and 65+ by social and religious resources: Health and Retirement Study, 2010/2012.

Figure 1 presents weighted mean levels of hopelessness by each resource for blacks in midlife (age 51–64 years old) and blacks in old age (age 65 and older). For both age groups, blacks with lower levels of each resource had significantly higher mean hopelessness levels as determined by the nonoverlapping confidence intervals comparing the high and low levels of each resource. This finding suggests that greater social support, social engagement, religious attendance, and religiosity may be protective against hopelessness for both age groups. Additionally, mean hopelessness levels for blacks with low levels of each resource did not differ significantly across the four resources for either age group, nor did mean hopelessness levels among blacks with high levels of each resource. These findings suggest potentially similar effects of each resource among both age groups.

Table 2 presents the regression of hopelessness on study variables for middle-aged blacks. More frequent reports of everyday discrimination were associated with greater hopelessness net of covariates (Model 1: β = 0.247; *p* < .001). Adding the social and religious resources slightly reduced but did not fully account for the effects of discrimination on hopelessness (Model 2). Individuals with high levels of social support (Model 1: β = −0.294; *p* < .01), religious service attendance (Model 2: β = −0.281; *p* < .05), and religiosity (Model 2: β = −0.297; *p* < .001) had lower hopelessness levels compared to individuals with low levels of these resources; social engagement was not a statistically significant predictor of hopelessness in this model. Next, we assessed the interaction between discrimination and social support (Model 3), social engagement (Model 4), religious attendance (Model 5), and religiosity (Model 6). None of the interaction terms was statistically significant.

**Table 2.** Weighted Linear Regression of Hopelessness on Everyday Discrimination and Social and Religious Resources among Blacks Age 51–64: Health and Retirement Study, 2010/2012 (*n* = 1,302)

	Model 1		Model 2		Model 3		Model 4		Model 5		Model 6	
	*b*	*SE*	*b*	*SE*	*b*	*SE*	*b*	*SE*	*b*	*SE*	*b*	*SE*
Everyday discrimination	0.247***	0.058	0.211**	0.062	0.215**	0.076	0.263*	0.113	0.222**	0.072	0.212**	0.072
Social support^a^			−0.294**	0.097	−0.287*	0.134	−0.291**	0.095	−0.294**	0.097	−0.294**	0.096
Social engagement^b^			−0.101	0.078	−0.100	0.078	−0.021	0.136	−0.101	0.078	−0.101	0.077
Religious service attendance^c^			−0.218*	0.100	−0.218*	0.100	−0.218*	0.099	−0.199	0.141	−0.218*	0.100
Religiosity^d^			−0.297***	0.080	−0.297***	0.081	−0.302***	0.080	−0.297***	0.080	−0.295**	0.109
Discrimination × Support					−0.008	0.082						
Discrimination × Engagement							−0.090	0.129				
Discrimination × Religious attendance									−0.022	0.114		
Discrimination × Religiosity											−0.002	.084

*Notes*: Models control for gender, marital status, nativity, employment status, education, poverty status, and depressive symptoms.

^a^Ref = low social support. ^b^Ref = low social engagement. ^c^Ref = attend religious service less than once a week. ^d^Ref = low religiosity.

*p < .05. **p < .01. ***p < .001.

**Figure 2.**
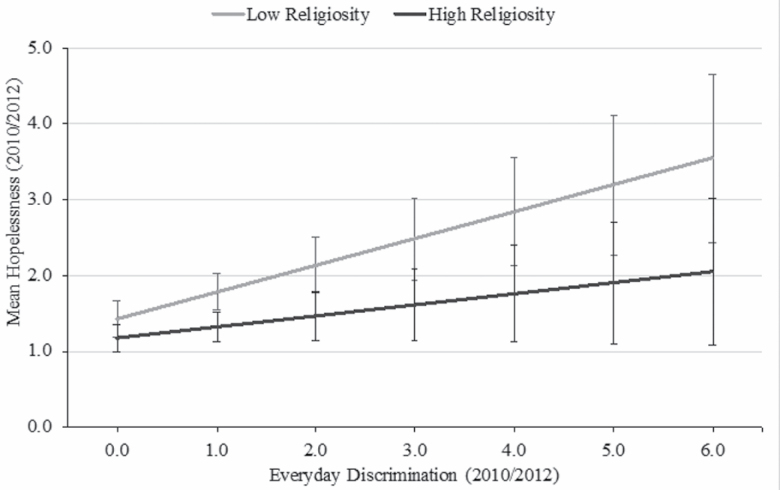
Predicted mean hopelessness by everyday discrimination and religiosity for blacks age 65+ (*n* = 887): Health and Retirement Study, 2010/2012.

The same regression models were assessed for older blacks (Table 3). Everyday discrimination was positively associated with hopelessness net of covariates (Model 1: β = 0.304; *p* < .001). Social support (Model 2: β = −0.304; *p* < .05), social engagement (Model 2: β = −0.236; *p* < .05), and religiosity (Model 2: β = −0.369; *p* < .05) were inversely associated with hopelessness; specifically, individuals with high levels of each resource had lower levels of hopelessness compared to those with low levels of the resource. Social support (Model 3), social engagement (Model 4), and religious attendance (Model 5) did not moderate the relationships between everyday discrimination and hopelessness. However, the interaction between discrimination and religiosity was statistically significant (Model 6: β = −0.208; *p* < .05). Specifically, older blacks with low levels of religiosity experienced steeper increases in hopelessness due to discrimination than older blacks with high levels of religiosity (Figure 2).

**Table 3.** Weighted Linear Regression of Hopelessness on Everyday Discrimination and Social and Religious Resources among Blacks Age 65+: Health and Retirement Study, 2010/2012 (*n* = 887)

	Model 1		Model 2		Model 3		Model 4		Model 5		Model 6	
	*b*	*SE*	*b*	*SE*	*b*	*SE*	*b*	*SE*	*b*	*SE*	*b*	*SE*
Everyday discrimination	0.304***	0.079	0.259**	0.080	0.211	0.129	0.326***	0.071	0.388**	0.130	0.353***	0.097
Social support^a^			−0.304*	0.143	−0.367*	0.153	−0.298*	0.142	−0.310*	0.142	−0.307*	0.142
Social engagement^b^			−0.236*	0.109	−0.241*	0.111	−0.158	0.104	−0.249*	0.108	−0.233*	0.105
Religious service attendance^c^			−0.108	0.078	−0.102	0.074	−0.116	0.079	0.018	0.114	−0.117	0.078
Religiosity^d^			−0.369***	0.103	−0.367***	0.102	−0.369***	0.104	−0.370***	0.101	−0.242*	0.104
Discrimination × Support					0.089	0.157						
Discrimination × Engagement							−0.120	0.109				
Discrimination × Religious attendance									−0.202	0.153		
Discrimination × Religiosity											−0.208*	0.091

*Notes*: Models control for gender, marital status, nativity, employment status, education, poverty status, and depressive symptoms.

^a^Ref = low social support. ^b^Ref = low social engagement. ^c^Ref = attend religious service less than once a week. ^d^Ref = low religiosity.

*p < .05. **p < .01. ***p < .001.

Supplemental analyses of perceived discrimination attributions revealed that 35% of respondents who report experiencing discrimination attribute their experiences to race alone, but nearly half reported some combination of race, gender, and age as the reason for these experiences (see Supplementary Table S1). Additionally, supplemental regression analyses including an indicator for whether respondents attributed their reported experiences of discrimination to race did not find a significant additive or multiplicative effect of this attribution on hopelessness or the discrimination–hopelessness relationship, respectively (see Supplementary Tables S2 and S3).

## Discussion and Implications

The current study revealed a significant positive association between discrimination and hopelessness among middle-aged and older blacks, which supports our hypothesis and aligns with prior research on the effects of discrimination on mental and physical health (72). The null findings from supplemental analyses examining race as a perceived reason or attribution for self-reported discrimination suggest that discrimination is harmful for middle-aged and older blacks regardless if they attribute these discriminatory experiences to their race, highlighting the importance of considering the intersecting identities of middle-aged and older blacks and the multiple types of discrimination they face. Counter to our hypotheses, social support from family and friends, religious involvement, and religiosity mitigated feelings of hopelessness but did not buffer the effects of discrimination on hopelessness for middle-aged blacks. For older blacks, social support, social engagement, and religiosity protected against hopelessness, but only religiosity buffered the effects of discrimination on hopelessness, which partially supports our hypotheses about the greater salience of religious resources for older blacks in our sample. Thus, social and religious resources tend to offset increases in hopelessness that result from experiencing discrimination, but which resources are protective and whether they specifically buffer the effects of discrimination on hopelessness may be specific to age groups among blacks.

We hypothesized that all of the resources examined would be protective against hopelessness regardless of age group. However, among the four resources, weekly religious service attendance was not protective for blacks in old age, and social engagement was not protective for blacks in midlife. Possible explanations for these findings are likely related to characteristics of the aging process and life course development. For instance, prior research suggests that religious attendance varies across the life course: declining in adolescence, increasing in early adulthood, remaining relatively stable in midlife, and then declining in older adulthood after a slight increase post-retirement (74). This decline in attendance during older adulthood may be due to the greater onset of health conditions and physical limitations that prevent older adults from regularly attending religious services (75). Given that a person is more likely to receive support from church congregants, a social resource, when they are physically present at church, the benefits of religious service attendance may be diminished among older adults because they are unable to attend religious services regularly. Additionally, older blacks who are able to attend church may be more likely to experience the death of similarly aged peers in their church support network, which can reduce the amount of support they receive relative to support received earlier in the life course (76). Consequently, older blacks may rely more heavily on their kin and religious beliefs for support and coping than their congregation.

For blacks in midlife, objective aspects of social engagement, specifically the frequency of contact with family members, neighbors, and friends, may play a lesser role in hopelessness than the subjective aspects of engagement, such as the quality of relationships and support received from different sources. Nguyen and colleagues (48) found that subjective closeness to one’s family and friends was more important for the happiness and life satisfaction of blacks aged 55 and older than the frequency of contact with family and friends. Relationship closeness and other qualitative aspects of social networks may be particularly important to blacks who have yet to reach retirement age because of potentially competing caregiving demands: caring for adult children and aging parents. Although blacks tend to view caretaking more favorably than whites (77), the time given to caring for others may limit their ability to have regular contact with members of their social network. Moreover, prior research has shown that negative interactions with family adversely affect well-being and can increase suicidal ideation and attempts (44). Thus, the quality of interactions with family and friends and the emotional support they provide may be more protective against hopelessness, even if those interactions are less frequent.

Social support and religiosity were inversely associated with hopelessness for both age groups. These findings align with research demonstrating the protective effects of both resources on mental health (78,79). Social support, however, did not buffer the effects of discrimination, as evident by the null finding for the discrimination–support interaction term. Although some studies have also found that emotional social support buffers the effects of discrimination on mental health (8), others have not (80). Our findings may align with a resource deterioration model for the relationship between social stressors and coping resources, such that social support functions as a mediator instead of a moderator of this relationship (38). The chronic stress of everyday experiences of discrimination may tax and erode the social networks from which blacks receive emotional support and, thus, leave them more vulnerable to the adverse effects of discrimination on mental health. Empirical evidence exists in support of the resource deterioration model for the discrimination–social support relationship (81) and in the current study the direct effect of discrimination on hopelessness was diminished by the inclusion of the social and religious resources in the regression model. Supplemental analyses suggest that the addition of social support was the primary driver of this reduction. Thus, rather than buffering the effects of discrimination on hopelessness among blacks, emotional social support from family and friends may be partially eroded by discrimination. A formal test of this hypothesis is beyond the scope of the current study but represents an important direction for future research.

Unlike the other resources, we found a significant interaction between everyday discrimination and religiosity, but only among older blacks. This finding is counter to our hypothesis that both weekly religious attendance and high religiosity would fully buffer the effects of discrimination on hopelessness; however, it provides some support for the protective role religiosity plays against discrimination. Prior research suggests that religiosity and similar constructs buffer the effects of discrimination on mental health. For instance, in a study of psychological distress among blacks, Ellison and colleagues (82) found a significant interaction between discrimination and religious guidance—the extent to which one’s religion provides guidance in day-to-day living. Although our religiosity measure does not assess guidance, one item asks about the extent to which religious beliefs are incorporated into one’s life. Thus, our findings for religiosity contribute to research on the stress-buffering role of religious resources.

Although stress buffering by religiosity only occurred among older blacks, this finding may be explained by the positive association between religiosity and age (83). Aging is associated with an increased awareness of the finality of life and more frequent experiences of death, including the death of a spouse or other family members and friends of similar age. Older blacks may therefore turn to religion to a greater extent as a way of coping with and finding meaning in these major life events (84). Additionally, blacks in our sample were born between 1912 and 1960 and a larger proportion of older blacks (i.e., those 65 and older) came of age during the Civil Rights Movement. During this time, the Black Church played a significant role in the everyday lives of black Americans, including offering messages of faith and resilience in the face of racism and adversity (49). Consequently, blacks who were in young adulthood and midlife during this time may more readily mobilize religious beliefs and practices to address present-day experiences of discrimination. These age and cohort effects may explain why religiosity specifically buffers the effects of discrimination on hopelessness among older blacks.

There are limitations to this study that should be considered when interpreting the study findings and addressed in future research. First, we stratified our analyses by age based on conceptual and empirical literature suggesting differences in the experiences, exposures, behaviors, and health of those in midlife compared to old age (85). However, supplemental analyses testing the conditional effects of self-reported discrimination and each of the social and religious resources based on age were not statistically significant at a .05 significance level. Thus, our findings do not provide evidence for age differences in the associations assessed. In addition, our analyses are based on self-reported data that are subject to social desirability and recall bias. Our measure of discrimination, in particular, is a self-reported measure and may not accurately capture the frequency of actual incidences of discrimination. For religiosity, we dichotomized the variable at its median, to create a more even distribution of respondents across low and high levels of the resource. Fifty percent of the sample had a religiosity score less than 5 (i.e., median value = 5), while the remaining 50% had a score equal to 5, which is the maximum value on the religiosity scale (i.e., 5). Thus, findings for the relationship between hopelessness and religiosity only reflect distinctions between the highest levels of religiosity and everyone else. This finding, however, indicates that middle-aged and older blacks are highly religious, which is consistent with a long tradition of religiosity research showing very high levels of religiosity among blacks as a population and older blacks in particular (86). An additional study limitation is that the data are cross-sectional, which precludes statements of causality. Feelings of hopelessness may lead to perceptions of a lack of support, limit the desire to engage socially or attend religious services, and reduce religious interest and religious beliefs. Moreover, hopeless individuals also may be more aware of or more likely to report experiences of discrimination and depressive symptoms. Therefore, future research using longitudinal data would be beneficial for clarifying the directionality of these relationships. Lastly, some research suggests that hopelessness can be considered either a transient state or a personal trait (87). The four items included in our hopelessness measure do not distinguish between these two types of hopelessness, which is beyond the scope of this study, and another area of future research.

There are multiple strengths to the current study, including the use of a large and nationally representative sample of blacks in midlife and old age, which improves the generalizability of our findings to the larger population of blacks in the “second half of life.” Additionally, stratifying our analyses by age acknowledges the heterogeneity in lived experiences among U.S. blacks and allowed for a more nuanced and specific examination of how discrimination affects hopelessness in the context of different types of resources aimed at offsetting and/or protecting against its deleterious effects. This study is one of the first to address this understudied area of research in a well-characterized, national sample of blacks. Lastly, but perhaps most importantly, this study identified modifiable factors that influence hopelessness in the black community. Prior research has shown that hope and hopelessness are modifiable risk factors for poor health (88) and we have shown that social and religious resources may protect against feelings of hopelessness. Therefore, interventions that increase social engagement and emotional support, facilitate religious service attendance, and foster greater religiosity among blacks in midlife and old age may mitigate hopelessness among a group of individuals chronically beset by discrimination.

## Conclusions

Despite its association with an increased risk for death and disease, hopelessness remains an understudied consequence of racism and discrimination. The current study showed that more frequent reports of discrimination increase risk for hopelessness among middle-aged and older blacks and that social and religious resources generally protect against poor mental health. Our study also highlights the importance of recognizing the heterogeneity of experiences and resources within the black population at different life stages to better understand how discrimination affects their health and what factors effectively protect against hopelessness and despair. To the extent that hopelessness and the resources examined in the current study are all modifiable factors, aging researchers and practitioners should work to identify policy, community, and church-based interventions that protect against hopelessness and ultimately improve the mental health and functioning of older members of the black community.

## Supplementary Material

igaa044_suppl_Supplementary_Tables_S1-S3
