# Supplementary material for: Hopelessness Among Middle-Aged and Older Blacks: The Negative Impact of Discrimination and Protecting Power of Social and Religious Resources
Source: Innov Aging. 2020 Sep 15;4(5):igaa044. doi: 10.1093/geroni/igaa044 (PMC7679998; doi:10.1093/geroni/igaa044)
Supplement: igaa044_suppl_Supplementary_Tables_S1-S3 [file igaa044_suppl_supplementary_tables_s1-s3.docx]

Online Supplementary Material for Accepted Manuscript in *Innovation in Aging*:

Mitchell UA, Gutierrez-Kapheim M, Nguyen AW, Al-Amin N. Hopelessness among middle-aged and older Blacks: the negative impact of discrimination and protecting power of social and religious resources. *Innov Aging*. 2020.

| Supplemental Table S1. Attributions of Everyday Discrimination Among Blacks in Sample: Health and Retirement Study, 2010/2012 (n=1,122)* | | |
| --- | --- | --- |
| Attribution | % | n |
| Race | 35.3 | 396 |
| Age | 12.4 | 139 |
| Gender | 2.8 | 31 |
| Race & Age | 18.8 | 211 |
| Race & Gender | 10.2 | 114 |
| Age & Gender | 2.0 | 22 |
| Race, Age & Gender | 18.6 | 209 |
| *1,067 persons did not report an attribution. | | |

| Supplemental Table S2. Weighted Linear Regression of Hopelessness on Everyday Discrimination, Racial Attribution, and Social and Religious Resources among Blacks Age 51-64: Health and Retirement Study, 2010/2012 (n=1,302) | | | | |
| --- | --- | --- | --- | --- |
|  | Model 1 | | Model 2 | |
|  | b | SE | b | SE |
| Everyday Discrimination | 0.223** | 0.064 | 0.261** | 0.086 |
| Social Support^a^ | -0.295** | 0.097 | -0.292** | 0.098 |
| Social Engagement^b^ | -0.099 | 0.078 | -0.102 | 0.077 |
| Religious Service Attendance^c^ | -0.218* | 0.100 | -0.215* | 0.099 |
| Religiosity^d^ | -0.298*** | 0.080 | -0.299*** | 0.080 |
| Race Attribution^e^ | -0.042 | 0.066 | 0.012 | 0.095 |
| Discrimination-x-Race Attribution |  |  | -0.066 | 0.093 |
| *Notes*. *p<0.05, **p<0.01, ***p<0.001; ^a^ref=low social support, ^b^ref=low social engagement, ^c^ref=attend religious service less than once a week; ^d^ref=low religiosity; ^e^ref=does not mention race. Models control for gender, marital status, nativity, employment status, education, poverty status, and depressive symptoms. | | | | |

| Supplemental Table S3. Weighted Linear Regression of Hopelessness on Everyday Discrimination, Racial Attribution, and Social and Religious Resources among Blacks Age 65+: Health and Retirement Study, 2010/2012 (n=887) | | | | |
| --- | --- | --- | --- | --- |
|  | Model 1 | | Model 2 | |
|  | b | SE | b | SE |
| Everyday Discrimination | 0.270*** | 0.076 | 0.256** | 0.095 |
| Social Support^a^ | -0.308* | 0.145 | -0.309* | 0.145 |
| Social Engagement^b^ | -0.230* | 0.107 | -0.233* | 0.102 |
| Religious Service Attendance^c^ | -0.109 | 0.077 | -0.110 | 0.077 |
| Religiosity^d^ | -0.372*** | 0.104 | -0.374*** | 0.105 |
| Race Attribution^e^ | -0.051 | 0.102 | -0.086 | 0.168 |
| Discrimination-x-Race Attribution |  |  | 0.041 | 0.187 |
| *Notes*. *p<0.05, **p<0.01, ***p<0.001; aref=low social support, bref=low social engagement, cref=attend religious service less than once a week; dref=low religiosity; ^e^ref=does not mention race. Models control for gender, marital status, nativity, employment status, education, poverty status, and depressive symptoms. | | | | |
